# Rectal Prolapse in the Pediatric Population

**DOI:** 10.1007/s11894-024-00953-5

**Published:** 2024-11-23

**Authors:** James K. Moon, John D. Stratigis, Aaron M. Lipskar

**Affiliations:** Northwell, Cohen Children’s Medical Center, Division of Pediatric General, Thoracic, and Endoscopic Surgery, Northwell Health, 2000 Marcus Ave, Suite 300, New Hyde Park, NY 11042-1069 USA

**Keywords:** Pediatric, Rectal prolapse, Colorectal, Surgery

## Abstract

**Purpose of Review:**

Rectal prolapse in the pediatric population presents a clinical challenge with wide variability in etiology, presentation, work-up and management. In this article, we reviewed the evidence supporting various medical and surgical treatment options as well as the recent trends amongst pediatric surgeons.

**Recent Findings:**

Medical therapy is highly effective in most patients, with bowel management programs being particularly successful. Nonetheless, medically refractory disease, often seen in older children and in children with behavioral/psychiatric disorders, can be challenging. Sclerotherapy with ethanol or 5% phenol can be effective local treatments. 15% hypertonic saline, 50% dextrose, and Deflux are additional safe alternatives. Perianal procedures and perineal procedures are less invasive surgical options, but transabdominal rectopexy appears to be the favored treatment for disease refractory to local treatment. Transabdominal rectopexy with sigmoidectomy, the recommended operation in the adult population for patients with prolapse and constipation, appears only to be preferred in the pediatric population for postoperative recurrences.

**Recent Findings:**

While outcomes of medical treatment for pediatric rectal prolapse are excellent, sclerotherapy and transabdominal rectopexy are effective options for refractory disease preferred by most pediatric surgeons.

## Introduction

Rectal prolapse is a condition of partial or full thickness circumferential protrusion of the rectal wall through the anus externally. While traditionally seen in elderly female patients with weakened pelvic floors, it is also commonly seen in the pediatric population. Most commonly rectal prolapse in children is encountered between infancy and 4 years of age. Children presenting after 4 years of age are thought to behave more like adults with rectal prolapse, with a lower success rate of medical management alone, thus more often requiring more aggressive surgical management [1, 2]. Incidence is similar between male and female children.

Pediatric rectal prolapse is thought to be secondary to an underlying predisposing condition, the most common of which is chronic constipation and stool withholding, most frequently seen during potty training years [3]. Other etiologies such as acute infectious diarrheal disease and intestinal parasites are more commonly seen in developing countries [4]. Congenital conditions associated with abnormal stooling such as myelomeningocele, spina bifida, Hirschsprung’s disease, anorectal anomalies, Ehlers-Danlos syndrome, and cystic fibrosis (CF) also predispose children to rectal prolapse [5]. CF in particular, was classically uniformly tested for in children that presented with rectal prolapse; however, with newborn screening for CF, routine testing in no longer recommended [6]. Patients with developmental delay and behavioral/psychiatric disorders present a unique challenge, as managing their stooling behaviors requires a multidisciplinary approach for best outcomes [5].

Children with rectal prolapse present to the office or emergency room with a mass protruding from the rectum. Most commonly, a circumferentially prolapsing mass covered with mucosa is seen initially by the child or the caregiver while straining, often during or after a bowel movement. The prolapse itself is painless but may induce discomfort and tenesmus. Passage of mucous is often seen and irritation of the prolapsed mucosa can also lead to bleeding. Workup of rectal prolapse starts with thorough history and physical and identifying the prolapse. If the prolapse is not easily appreciated during physical exam, maneuvers such as Valsalva, squatting, or attempts at defecation in the office can help provoke the prolapse. If the prolapse is not easily reproducible, parents are encouraged to photograph the prolapse when they see it at home. Once the prolapse is identified, the workup involves identifying the underlying predisposing condition. We routinely perform a contrast enema to identify the underlying stool burden as well as to rule out any anatomic abnormalities. Adjuncts such as MR defecography, video defecography, anal manometry, and proctosigmoidoscopy may be performed, especially if polyps or solitary rectal ulcers are suspected, but are not routinely used in children [7–9]. It is also important to rule out other diagnosis such as ileocecal intussusception, prolapsing rectal polyps, and prolapsing hemorrhoids.

The mainstay of treatment of pediatric rectal prolapse is to address the underlying predisposing condition. Nonetheless, frequent recurrent prolapse may require additional procedural and surgical interventions. While there lacks consensus among pediatric surgeons on the best practice treatment for rectal prolapse, we will review some of the more commonly performed procedures and supporting evidence.

## Treatments

### Medical Management

Medical treatment of rectal prolapse starts with reduction of the prolapsed rectum. While spontaneous reduction is common, manual reduction of prolapse may be needed at home, in the office, or in the emergency department. Manual reduction should be attempted with well lubricated gloved hands, with a finger placed in the rectum to guide the reduction, and steady constant pressure to reduce the prolapsed rectum into the anus. Prolonged prolapse may result in a more edematous rectum and adjuncts such as table sugar or 50% glucose solution may be applied to the rectal mucosa to reduce the edema and aid in prolapse reduction [10, 11]. After reduction of the prolapse, a pressure dressing around anus or taping the gluteal muscles can be used as a temporary measure to prevent immediate recurrence. While not easy, the child should try to relax and stay calm during the process as this will aid in allowing the prolapse to reduce and in helping to keep the prolapsed rectum reduced once in.

The focus of conservative management of rectal prolapse aims to address the underlying issue. In the absence of infectious or anatomical abnormalities, the preferred medical treatment modality is a bowel management program that provides a comprehensive protocol for treating constipation and promoting healthy stooling hygiene. While bowel management programs take many forms throughout the country, common recommendations include adequate fluid and fiber intake, good toileting habits, and stool softener/laxative therapy. Good toileting habits are important in reducing time on the toilet and straining. Patients are encouraged to spend no more than 5 min at a time on the toilet and to remove distractions such as mobile phones or other electronics during defecation. Good anatomical positioning with a step stool also promotes proper stooling and reduces straining. The choice of stool softener/laxatives may vary widely; however, stimulant laxatives such as Ex-lax and stool softeners such as Polyethylene glycol are commonly used.

High treatment success rates have been reported with medical management and bowel management programs [12]. One protocol included:

1. Patient education on good toileting habits.
2. High dose stimulant laxatives (Ex-lax, senna) dosed at 2 mg/kg/day and titrated to a goal of 1–2 soft bowel movements per day.
3. One week of daily abdominal radiographs and clinical nursing staff follow up.
4. Routine 6-month follow up [13].

In reviewing their outcomes from 2011 to 2022, Short et al. reported a 94% success rate in treating rectal prolapse with conservative management with a median time to resolution of 9-months.

### Sclerotherapy

While medical management success rates are high, refractory disease can be seen especially in children over 4 years of age, and in patients with behavioral/psychiatric disorders [2]. Determining the next steps in treatment can be quite difficult as there is wide variability in practice patterns, evidenced by over 40 procedures reported in the literature [14–17]. Duration of medical management is also not standardized before next steps are considered; however, a 2019 American Pediatric Surgery Association survey study reporting practice patterns amongst North American providers reported that 47.9% of providers would trial medical management for three to six months, while 28.1% of providers would continue medical management up to one year [18]. The same study reported that a majority of providers would consider sclerotherapy as the next step in management in rectal prolapse. The role of sclerotherapy in the treatment algorithm for adolescents and young adults is unclear, but there are minimal data to help guide these decisions.

Sclerotherapy is performed by injecting a sclerosing agent into the rectal submucosa above the dentate line. A circumferential submucosal wheel is made with the sclerosing agent taking care to avoid the anterior midline to avoid the genitourinary system. It is an outpatient procedure that requires minimal sedation and has an acceptable complication profile. Rectal sclerotherapy is thought to induce inflammation in the rectal submucosal tissue, which leads to scarring and bulking of the rectal wall to prevent prolapse [19]. Overall success rate of sclerotherapy varies widely from 29 to 96% depending on the sclerosing agent and number of injections [16]. The most commonly reported agents include ethanol and phenol [20], while 15% hypertonic saline, 50% dextrose (D-50), and Deflux use are also reported.

Ethanol protocols include 1.5 to 2 ml of 96% or 98% ethyl alcohol injections. Success rates have been reported between 70 and 89%, with complications of mucosal ulceration and rectovaginal fistula, temporary fecal soilage seen in 8–16% [21, 22].

5% phenol in peanut or almond oil has been used with a high success rate ranging from 65 to 100% [21, 23]. A typical protocol injects 0.5 ml/kg of total sclerosing agent into the submucosa. Despite its high success rate, high complication rates have also been reported, with up to 27% of patients experiencing issues with mucosal sloughing, perianal fistulas, and abscess formation [21], and a case report of death following phenol toxicity [24].

15% hypertonic saline between 2 ml to 10 ml was used to achieve a success rate of 71–94% with no reported complications in two studies [25, 26]. A more recent randomized control trial comparing 5% phenol with almond oil vs. 15% hypertonic saline treatment showed a superior success rate (50% in phenol vs. 77% in 15% hypertonic saline) and a low complication rate (2% incontinence, 2% anal stenosis) [27].

50% dextrose at a volume of 1 ml/kg has been reported to have a 93% success rate with minimal complications (1 case of self-limited bleeding) [28]. At our institution we use D-50 and have been satisfied with the outcomes.

Deflux (Q-Med, Uppsala, Sweden), is a polysaccharide mixture of hyaluronic acid (HA) and dextranomer (Dx) typically used for pediatric vesicoureteric reflux with an established long term safety profile [29]. It has also been used as a sclerosing agent for rectal prolapse. 1 cc total of premixed Deflux (50 mg/ml Dx, 15 mg/ml HA) injected into the submucosa has been shown to have a 100% success rate with no complications in a group of 28 patients [21]. One technical disadvantage of the Deflux protocol is the low total volume of sclerosing agent makes complete circumferential injections more difficult.

Another advantage of sclerotherapy is that it can be used more than once. 72% of practitioners in the 2019 APSA survey study responded that they would try up to three sessions of sclerotherapy before considering additional more invasive treatment options [18].

### Perianal Procedures

Perianal operative procedures such as Thiersche’s anal cerclage, Ekehorn’s Rectosacropexy, and Lockhart Mummery operation are described as local, less invasive procedural treatment options.

Thiersche’s anal cerclage involves placing a tunneled circumferential suture under the skin around the anal opening at the mucocutaneous junction. A finger or Hegar dilator is used to ensure adequate anal opening size. Success rate up to 90% have been reported.

Ekehorn’s Rectosacropexy is performed by placing a horizontal mattress suture in the rectal ampulla from the inside of the rectum through the lower part of the sacrum and out through at the skin and tied off externally to pexy the rectal wall and cause inflammation around the surrounding tissue. A braided silk suture is used then removed after 10 days. 100% success rates have been reported without major complications [14, 30].

The Lockhart Mummery operation is performed with a mesh gauze packing placed temporarily in the retrorectal space through a posterior perianal incision and was also performed in the past with high reported success rates [12].

Many modifications have been reported to perianal procedures with small series of favorable outcomes; however, these procedures have fallen out of favor given the effectiveness of sclerotherapy. Only 8.4% of providers would offer perianal procedures in the 2019 APSA survey, and most providers would consider proceeding directly to more invasive surgical options if sclerotherapy fails.

### Perineal Procedures

Posterior sagittal rectopexy is performed via a posterior sagittal midline incision with the patient in prone position. The incision is carried down to the level of the rectum. The rectum is identified with the aid of a Hegar dilator within the lumen, and the sides of the rectum are freed from the surround tissue. The dilated rectum is plicated and incorporated into the reapproximation of the levator ani and muscle complex posteriorly [31, 32]. The procedure is well tolerated, and can even be performed on an outpatient basis; however, the reported recurrence rate was as high as 70% [33].

Perineal proctectomy or proctosigmoidectomy was the preferred surgical approach after failure of medical management in 23% of providers in the 2019 APSA survey. While mostly described in the adult literature for prolapse, the perineal proctectomy and proctosigmoidectomy is a familiar surgery to pediatric surgeons who treat Hirschsprung’s disease. The redundant rectum and sigmoid are mobilized then resected through a full thickness incision above the dentate line and a mucosa to mucosa coloanal anastomosis is formed. Specific data for outcomes of perineal proctectomy and proctosigmoidectomy for pediatric rectal prolapse are lacking in the literature. There are, however, rare case reports such as a linear stapled modification of the Delorme’s procedure used in children [34].

### Transabdominal Rectopexy

Transabdominal rectopexy can be performed in an open, laparoscopic, or most recently, robotic fashion. The rectum is dissected towards the pelvis in the mesorectal plane then straightened out and fixed to the presacral fascia with a suture or laparoscopic tacks [35]. Transabdominal rectopexy outcomes vary with recurrence rates between 0 and 40% [2]. Nonetheless, direct comparisons between laparoscopic suture rectopexy and posterior sagittal rectopexy showed a 0% (0/6 patients) recurrence vs. 25% (2/8 patients) recurrence [36], and APSA providers from the 2019 survey also showed a preference for trans-abdominal rectopexy (66% providers) vs. perineal procedures (34% providers) [18].

Previously laparoscopic rectopexy in children has been shown to be an effective and safe option when compared to the traditional open rectopexy [37]. More recently, a prospective randomized study comparing robot-assisted vs. laparoscopic rectopexy in adults showed equivalent outcomes in operative time, length of stay, post-operative complications, and restoration of anatomy post-operatively seen on MR defaecography [38]. One retrospective pediatric study reviewed their robotic rectopexy experience with children 13 to 17 years of age. The average operative time was 347 min, and the average length of stay was 3.25 days. At a mean 11.5 month follow up, there were no recurrences noted [39].

One area of controversy is the role of concomitant resection (usually sigmoidectomy) at the time of rectopexy (“resection-rectopexy”). Constipation is the most significant complication post operatively after transabdominal rectopexy. Some limited data shows improved outcomes in terms of constipation with a resection at the time of rectopexy, but recurrence rates remain largely unchanged [37, 40]. In the adult literature, the 2017 American Society of Colon and Rectal Surgeons (ASCRS) rectal prolapse management guidelines gave a strong recommendation for rectopexy based on high quality evidence (1 A); Resection-rectopexy was only recommended in patients with baseline constipation based on moderate quality evidence (1B) due to concerns with worsening incontinence postoperatively [41]. Amongst pediatric surgeons, more APSA providers preferred transabdominal rectopexy over resection-rectopexy (45% vs. 21%) as the initial surgical approach after failure of medical management in all patients [18]; However, the preference for transabdominal resection-rectopexy increased dramatically when asked about preferred surgical approached after postoperative recurrence. 60% of providers chose a transabdominal resection and redo-rectopexy as their procedure of choice in recurrent disease after surgery [18].

An additional point of debate is the use of mesh as an adjunct to rectopexy. A laparoscopic modified Orr-Loygue mesh rectopexy has been described in children to be effective in treating recurrent rectal prolapse [42]. A recent prospective randomized trial reported improved outcomes in recurrence (0% in mesh rectopexy group vs. 14.2% in suture rectopexy group) with no difference in complication rates at 36 months follow up [43]. In the adult literature, ventral mesh rectopexy has started to gain favor as the procedure of choice given the limited posterior dissection with similar recurrence rates [41, 44]; However, potential longer term complications such as bowel obstruction and mesh erosion remain a concern and 70% of APSA surgeons performed rectopexy without a mesh routinely [18].

## Conclusions

Rectal prolapse in children remains a challenging problem with wide variability in treatment. Most patients, especially those younger than four years of age, will have self-limiting disease if compliant with appropriate medical management; however, older children and children with behavioral and psychiatric disorders can be particularly challenging in treating medically. Formal bowel management programs can be particularly effective in medically treating rectal prolapse. In refractory cases, first line of treatment after an adequate trial of medical management is most often sclerotherapy. While a wide range of sclerosing agents have been reported, ethanol and 5% phenol have been studied the most. The treatments can be highly effective, but complications from the agents have also been reported. While studies are more limited, 15% hypertonic saline, 50% dextrose, and Deflux may be effective and safe alternatives. Perianal procedures have been used as an adjunct or alternate to sclerotherapy. Thiersche’s anal cerclage, Ekehorn’s Rectosacropexy, and Lockhart Mummery operation are local procedures performed traditionally; however, more recently these procedures are less favored by pediatric surgeons. Perineal operations such as posterior sagittal rectopexy and perineal proctectomy/proctosigmoidectomy are more aggressive treatments for disease refractory to local therapy. While these surgeries are less invasive compared to transabdominal rectopexy, fewer surgeons prefer it due to higher reported recurrence rates. The most frequently performed surgery after failed local therapy is transabdominal rectopexy. Laparoscopic rectopexies are commonly performed with good outcomes, and recent advances in robotic approaches also show similar outcomes. Adjuncts such as sigmoidectomy or use of mesh at the time of rectopexy remain controversial. Nonetheless, transabdominal rectopexy with sigmoidectomy appears to be a consensus choice for recurrent disease after postoperative recurrence. While overall outcomes for pediatric rectal prolapse are excellent, further studies are required to optimize treatment for medically and surgically refractory disease.

## Key References

• Short SS, Wynne EK, Zobell S, et al. Most children experience resolution of idiopathic pediatric rectal prolapse with bowel management alone. J Pediatr Surg 2022;57(10):354-8.

o Well organized study of the effectiveness of a formal bowel management program in conservatively managing rectal prolapse. The group’s protocol is described in detail in the manuscript.

• Zhou W, Shi Y, Zhang M, et al. The Remission Effects of First Injection of Sclerotherapy for Pediatric Rectal Prolapse: A Systematic Review and Meta-Analysis. Front Surg 2022;9:835235.

o Meta-Analysis of sclerotherapy for pediatric rectal prolapse that extensively reviews the current literature. Summarizes and references each study in detail with specific injection agent and recurrence rate. Provided a framework for the injection sclerotherapy section of this manuscript.

• Trappey AF, 3rd, Galganski L, Saadai P, et al. Surgical management of pediatric rectal prolapse: A survey of the American Pediatric Surgical Association (APSA). J Pediatr Surg 2019;54(10):2149-54.

o Excellent survey study outlining the current trends and practices from a large group of American pediatric surgeons. The survey results were extensively discussed within the manuscript to compare current research and clinical practices.

• Mustafa G, Asad A, Tul Muntaha S. Comparison of 5% Phenol With Almond Oil Versus 15% Hypertonic Saline in Treatment of Pediatric Idiopathic Rectal Prolapse. Cureus 2022;14(3):e23552.

o One of the latest published randomized control trials comparing sclerotherapy agents for pediatric rectal prolapse.

• Yehya A, Gamaan I, Abdelrazek M, et al. Laparoscopic Suture versus Mesh Rectopexy for the Treatment of Persistent Complete Rectal Prolapse in Children: A Comparative Randomized Study. Minim Invasive Surg 2020;2020:3057528.

o Most recent prospective randomized control trial directly comparing laparoscopic suture rectopexy vs. mesh rectopexy in children.

## Data Availability

No datasets were generated or analysed during the current study.
